# Genetic dissection of cassava brown streak disease in a genomic selection population

**DOI:** 10.3389/fpls.2022.1099409

**Published:** 2023-01-13

**Authors:** Leah Nandudu, Robert Kawuki, Alex Ogbonna, Michael Kanaabi, Jean-Luc Jannink

**Affiliations:** ^1^ Section of Plant Breeding and Genetics, School of Integrative Plant Sciences, Cornell University, Ithaca, NY, United States; ^2^ Root crops Department National Crops Resources Research Institute (NaCRRI), Kampala, Uganda; ^3^ US Department of Agriculture, Agricultural Research Service (USDA-ARS), Ithaca, NY, United States

**Keywords:** cassava brown streak disease, incidence, severity, genome wide approach, susceptibility

## Abstract

**Introduction:**

Cassava brown streak disease (CBSD) is a major threat to food security in East and central Africa. Breeding for resistance against CBSD is the most economical and sustainable way of addressing this challenge.

**Methods:**

This study seeks to assess the (1) performance of CBSD incidence and severity; (2) identify genomic regions associated with CBSD traits and (3) candidate genes in the regions of interest, in the Cycle 2 population of the National Crops Resources Research Institute.

**Results:**

A total of 302 diverse clones were screened, revealing that CBSD incidence across growing seasons was 44%. Severity scores for both foliar and root symptoms ranged from 1.28 to 1.99 and 1.75 to 2.28, respectively across seasons. Broad sense heritability ranged from low to high (0.15 - 0.96), while narrow sense heritability ranged from low to moderate (0.03 - 0.61). Five QTLs, explaining approximately 19% phenotypic variation were identified for CBSD severity at 3 months after planting on chromosomes 1, 13, and 18 in the univariate GWAS analysis. Multivariate GWAS analysis identified 17 QTLs that were consistent with the univariate analysis including additional QTLs on chromosome 6. Seventy-seven genes were identified in these regions with functions such as catalytic activity, ATP-dependent activity, binding, response to stimulus, translation regulator activity, transporter activity among others.

**Discussion:**

These results suggest variation in virulence in the C2 population, largely due to genetics and annotated genes in these QTLs regions may play critical roles in virus initiation and replication, thus increasing susceptibility to CBSD.

## Introduction

As one of the world’s major food crops, cassava (*Manihot esculenta Crantz*) provides the third largest source of calories after maize and rice. The large starchy roots and edible leaves provide food for more than 800 million people (Nassar and Ortiz, 2010), most of whom are in sub-Saharan Africa. The crop produces reasonable yield in low agro-input farming systems under marginal soils and climatic conditions which makes it a decent food security crop with increasing global production. Cassava food products include boiled cassava, bread, pasta, noodles, cakes, and flour among others (Bechoff et al., 2018). Most of these products are crucial for sustainable food systems, in Africa and Latin America. High starch content in cassava tubers also makes the crop a suitable raw material for industrial applications like starch production, paper, plywood and veneer adhesives, alcohol, glucose, dextrin syrups, and biofuels among others (Lu et al., 2011; Ademiluyi and Mepba, 2013). The expected growth and boom in the cassava industry has made cassava a strategic crop for many governments particularly in Africa because this holds the key to creation of employment opportunities thus increasing incomes for better livelihoods.

In the last 90 years (Tomlinson et al., 2018), cassava production has been threatened by biotic stresses that are now elevated by climate change (Jarvis et al., 2012). Among these are cassava diseases, including cassava mosaic disease (CMD) and cassava brown streak disease (CBSD), that can cause up to 100% yield losses in susceptible varieties (Hillocks and Jennings, 2003). CMD is caused by cassava mosaic begomoviruses which are monopartite circular DNA viruses in the genus *Begomovirus* and family *Geminiviridae* (Walker et al., 2022), and are vectored by whiteflies. CMD is widespread in Africa and is caused by 11 viral species, 9 of which are from Africa (Patil and Fauquet, 2009). Breeding for resistance against CMD has led to the identification and deployment of CMD resistant varieties with both quantitative and recessive resistance from *Manihot glaziovii* (Thresh and Cooter, 2005; Fondong, 2017) or qualitative and dominant resistance from the CMD2 gene (Akano et al., 2002; Rabbi et al., 2014; Wolfe et al., 2016; Le et al., 2021). However, the same success has not been reported for CBSD because no known durable resistance genes or varieties have been identified and deployed.

CBSD is caused by a positive sense single-stranded RNA virus in the genus Ipomovirus and family *Potyviridae* (Winter et al., 2010; Walker et al., 2022) and is caused by two distinct viruses: cassava brown streak virus (CBSV) and Uganda cassava brown streak virus (UCBSV). Both viral species are collectively referred to as cassava brown streak viruses (CBSVs) and are vectored by whiteflies in a semi-persistent manner (where the virus is carried in the vectors’ guts but not spread to the salivary glands) in addition to the movement of infected stem cuttings by farmers (Maruthi et al., 2005; Mero et al., 2021). Genomes of both viruses are encoded as a single polyprotein that is autocatalytically cleaved into 10 mature proteins with sizes ranging between 8.9 to 10.8kb (Winter et al., 2010). CBSV has more non-synonymous substitutions in nucleotides across the genome compared to synonymous substitutions (Alicai et al., 2016) and is genetically more diverse with a large genetic landscape compared to UCBSV. This gives an advantage to CBSV in adapting to host changes and even overcoming host immune responses. It is also reported that CBSV genes like P1, 6K2, NIb and NIa have accelerated evolution rates (Alicai et al., 2016). Despite these differences at the molecular level, CBSVs have comparable foliar and root symptoms that start as leaf chlorosis along secondary vein margins developing into blotches. This is then followed by brown streaks on stems, radial root constrictions and root necrosis (Hillocks and Jennings, 2003; Alicai et al., 2007; Kaweesi et al., 2014). Root necrosis is the most devastating symptom because it renders the roots, which are of great economic value, inedible to both man and animals. For this reason, CBSD has been ranked among the seven most serious threats to world food security (Pennisi, 2010).

The development and deployment of CBSD resistant varieties remains the most effective and sustainable way of controlling CBSD. Breeding for resistance against CBSD has become a priority for cassava breeding programs in affected regions of East and Central Africa, and pre-emptive breeding for West Africa, a region not yet affected, is underway (Ano et al., 2021). Since the discovery of CBSD in the 1930’s in Tanzania, low but acceptable genetic gains have been attained through recurrent selection with the Amani inter-specific clones like Namikonga (also known as Kaleso or No.46106/27) and Kiroba as CBSD resistance donors (Nzuki et al., 2017; Masumba et al., 2017). These Amani inter-specific clones were created by crossing landraces with wild cassava (*Manihot glaziovii*, *Manihot dichotoma*, *Manihot catingae*, *Manihot melanobasis* and *Manihot saxicola*) (Hahn et al., 1980). The low genetic gains in cassava breeding are partly due to breeding complexities like variable flowering patterns, low seed set, low germination rates, long cropping cycles (12-14 months) and low multiplication rate of planting material (Ceballos et al., 2012; Ceballos et al., 2021) which makes it difficult to breed cassava in general. The lack of known durable/high sources of resistance in African breeding populations also makes it specifically difficult to breed for CBSVs resistant varieties (Sheat et al., 2019).

Rapid advances in next-generation sequencing (NGS) and statistical methods have created a platform for implementing modern breeding techniques like marker assisted selection (MAS) and genomic selection (GS) in cassava breeding. Large investments have been made to implement GS which predicts quantitative traits that are often expensive to phenotype using DNA markers across the genome (Meuwissen et al., 2001). The ability to estimate genomic-estimated breeding values (GEBVs) of new clones reduces phenotyping costs, increases selection intensity, enriches positive alleles in populations and shortens breeding time (Lehermeier et al., 2017). GEBVs also enable sparse testing that reduces the number of multi-environments breeding trials, further underscoring cost reduction and increase in testing capacity (Jarquin et al., 2020). Despite this genomic boom, few studies have been implemented in dissecting the genetic architecture of CBSD, identifying molecular markers and candidate genes associated with CBSD traits (Maruthi et al., 2014; Nzuki et al., 2017; Masumba et al., 2017; Amuge et al., 2017; Kayondo et al., 2018) compared to other crops like corn and rice. For instance, two genomic regions on chromosomes 4 and 11 were associated with CBSD foliar symptoms by (Kayondo et al., 2018) using genome wide association studies (GWAS). Nucleotide-binding site leucine rich repeat (NBS-LRR) genes that are known to play a role in disease resistance were associated with the chromosome 11 GWAS hit. Similar observations were made by (Kawuki et al., 2016) whose study identified seven significant SNP (single nucleotide polymorphisms) markers on chromosome 11 associated with mean root severity and disease index data.

Other studies have used biparental populations (Nzuki et al., 2017; Masumba et al., 2017) and have identified quantitative trait loci (QTLs) associated with CBSD symptoms. Nine QTLs on chromosomes 4, 5, 6, 11, 12, 15, 17, and 18 (Nzuki et al., 2017) were identified with different QTLs associated with CBSD foliar symptoms and root necrosis. Likewise, three QTLs on chromosomes 2, 11 and 18 were associated with CBSD foliar and root symptoms by (Masumba et al., 2017). 27 annotated genes were identified on chromosome 18 that code for Leucine Rich Repeat (LRR) proteins and signal recognition particles (Masumba et al., 2017). Comparing all these studies shows that numerous QTLs have been associated with CBSD foliar and root symptoms which confirms that that CBSD is a quantitative trait (Kayondo et al., 2018) that is controlled by polygenes with small effects, and these are often difficult to consistently identify (Wang et al., 2016; Wen et al., 2019). Despite identifying these QTLs, none of them have been validated as markers for use in CBSD breeding programs.

The National Crops Resources Research Institute (NaCRRI) in Uganda was one of the first African cassava breeding programs to implement genomic selection (GS) for routine breeding of traits with economic importance (Ozimati et al., 2018; Ozimati et al., 2019). Through GS, the baseline population Cycle 0 (C0) and the subsequent C1 population were developed and characterized for CBSD and other yield related traits (Ozimati et al., 2018; Kayondo et al., 2018; Ozimati et al., 2019). Subsequently, the cycle 2 (C2) population was developed in 2016/2017 and requires the characterization of CBSD traits. Therefore, this study seeks to highlight the impact of genomic selection in CBSD resistance breeding by characterizing the performance of the C2 population for CBSD incidence and severity in addition to identifying genomic regions and candidate genes associated with these CBSD traits.

The specific objectives are (1) evaluate CBSD trait variability in the C2 population, (2) establish phenotypic and genotypic correlations of CBSD traits and (3) identify genomic regions associated with CBSD traits in univariate and multivariate GWAS analyses to guide marker development for routine breeding, and (4) provide information on the functional annotated genes in the GWAS regions of interest. The results from this study will add to the existing knowledge especially on the genetic architecture of CBSD, providing insights that will be leveraged in breeding for resistance against cassava brown streak viruses.

## Materials and methods

### Plant material and field conditions

The cycle two (C2) population of genomic selection was developed at the National Crops Resources Research Institute, Uganda. It incorporated two clonal evaluation trials (CETs) that were planted in two locations in 2019/2020 and 2020/2021. Briefly, the C2 population resulted from successive cycles of selection and hybridization of clones selected based on genomic estimated breeding values (GEBVs) from the cycle zero (C0) and cycle one (C1) populations (Ozimati et al., 2018; Ozimati et al., 2019). Ninety-five (95) clones were selected from the C1 population and were hybridized to create 6,570 seedlings. These seedlings were planted in an unreplicated trial in Namulonge and were naturally infected with CBSD using whiteflies with spreader rows of TME204 as the source of inoculum. At harvest, 302 seedlings that had no visible CBSD symptoms and were vigorous enough to provide adequate planting material for the CETs were selected.

CETs were established in Serere and Namulonge in an augmented incomplete block design with three check varieties (UG110017, TME204 and Mkumba) planted in each block. Each plot was made up of ten plants that were planted in a single row with 1m spacing both within and between rows. Spreader rows of TME204 were also included in the CETs to increase disease pressure across both environments. These environments are associated with high CBSD disease pressure, mixture of both viruses and ‘superabundant’ whitefly populations (Alicai et al., 2007; Kawuki et al., 2016; Ally et al., 2019). Namulonge is located at a mid-altitude elevation of 1150 m above sea level (masl) with a bi-modal annual rainfall pattern of 1270 mm and a mean temperature of 22.2°C. Soils at this experimental site are characterized as red sandy clay loam with a pH of 4.9-5.0. Serere is located at 1140 masl with low annual rainfall of 900-1300mm and annual average temperature of 26°C. The soil is a sandy loam with a pH of 5.2-6.0. No agrochemicals or fertilizers were added to the trials.

### CBSD field evaluations

We used the 1-5 visual scoring scale (Legg and Thresh, 1998) for both CBSD foliar and root symptoms to assess disease severity at 3, 6 and 12 months after planting (MAP). CBSD foliar severities determined at 3 and 6 MAP were based on symptom expression on the leaves and stems, while root severity scores evaluated at 12MAP were based on the proportion of necrotic lesions in relation to the area of the cross-sectionally sliced root discs as described by (Masumba et al., 2017). CBSD foliar incidence was recorded as a percentage obtained from the number of plants that showed symptoms divided by the total number of plants in a plot while CBSD root incidence was obtained by dividing the number of roots that showed symptoms by the total number of roots in a plot.

### DArTseq genotyping

Two young top leaves were collected from each seedling of interest, folded, punched using a 5mm hand puncher and placed in 96-well plates. DNA extraction, Genotyping-by-Sequencing and SNP calling were carried out for each sample using DArTseq genotyping platform (https://www.diversityarrays.com/technology-and-resources/dartreseq/). A total of 28,434 markers were called and these were combined with another imputed genotype dataset that consisted of common SNPs between DArTseq and GBS sequencing platforms (obtained from Marnin Wolfe, unpublished data) bringing the SNPs to 51,865. Combining both marker datasets improved SNP coverage. To increase the association power and account for the possibility of sequencing error, an additional filtering step was performed on the combined marker dataset to remove genotypes with >10% and SNPs with >5% missing data or with minor allele frequency of less than 5%. A total of 30,846 SNP markers were obtained after filtering and for downstream analyses, SNP markers were converted to the dosage format of 1, 0, -1, which represented alternative allele homozygotes, heterozygotes, and reference allele homozygotes, respectively.

### Statistical analyses

#### Broad-sense and narrow sense heritability

Two linear mixed effects models were fitted using *lme4* package in R (R Development Core Team, 2016):

y_ijc_ = μ_i:c_ + g_i:c_ + β_j_ + r_i:c(j)_ + ϵ_ij_ Full model

y_ijc_ = μ_i:c_ + g_i:c_ + β_j_ + ϵ_ij_ Reduced model

Where y_ijc_ was a vector of phenotypic data, μ_i:c_ were fixed effects for the three checks and the population mean of the experimental clones with *i* indexing the checks and *c* indicating whether y_ijc_ is a check or an experimental clone. g_i:c_ are random effects of genotypes i with g_i_ ~ N (0, $\sigma_{\text{g}}^{2}$ ); β_j_ are random effects of year-location-incomplete block combination j with β_j_ ~ N (0, $\sigma_{\beta }^{2}$ ); r_i(j)_ are random effects of genotypes nested within year-location-incomplete block combination assumed to have a distribution of r_i:c(j)_ ~ N (0, $\sigma_{\text{r}}^{2}$ ); and ϵ_ij_ is the residual with ϵ_ij_ ~ N (0, $\sigma_{\text{e}}^{2}$ ). Variances were partitioned, and broad sense heritability was calculated by as H^2^ = σ _gi:c_^2^ / [σ _gi:c_^2^ + σ _ri:c(j)_^2^ + ${\text{σ}}_{\text{ϵij}}^{2}$ ]; where σ_gi:c_^2^ was the genotypic variance, σr_i:c(j)_^2^ variance of genotypes nested within the year-location-incomplete block combination and ${\text{σ}}_{\text{ϵij}}^{2}$ was model residual variance.

Narrow sense heritability was estimated using the function emmreml in the *EMMREML* package (Akdemir and Okeke, 2015) in R.

y = μ + Z_i:c_ a + Z_j_ b + Z_i:c(j)_ c + ϵ_ij_

where y represented the phenotypic data, vector a and the corresponding Z matrix represented random effects of genotypes with a distribution of a ~N (0, Kσ_a_^2^), K is the kinship matrix. Vector b and the corresponding Z matrix represented year-location-incomplete block combination with a distribution of b ~ N (0, Iσ_b_^2^). Vector c and the corresponding Z matrix represented genotypes nested in year-location-incomplete block combination with a distribution of c ~N (0, I_4_⊗K $\sigma_{\text{c}}^{2}$ ) while ϵ_ij_ was the residual with a distribution of ϵ_ij_ ~N (0, I $\sigma_{\text{e}}^{2}$ ). Narrow sense heritability was calculated using h^2^ = σ _Zi:c_^2^/ [σ _Zi:c_^2^ + ${\text{σ}}_{\text{ϵij}}^{2}$ ]; where σ _Zi:c_^2^ was additive variance and $\sigma_{\text{ϵij}}^{2}$ was the model residual variance. In addition to heritability estimates, descriptive statistics of mean, standard deviation, minimum and maximum values of all CBSD traits in the C2 population were determined using the mean, standard deviation, minimum and maximum functions in R.

### Trait correlations

Trait correlations of CBSD incidence and severity traits at 3, 6 and 12 MAP (CBSDi3, CBSDi6, CBSDRi, CBSDs3, CBSDs6, and CBSDRs) were evaluated based on phenotypic values, BLUPs, and GEBVs. All analyses were performed using the *cor* function in R package (R Development Core Team 2016), and visualization of the correlation matrices was done using the ‘*corrplot*’ R package (Wei and Simko, 2017).

### Two stage genome wide association study

In the first stage of genomic analysis, deregressed BLUPs were calculated from BLUPs obtained in the full model using the formula


$$deregressed\;BLUP=\frac{BLUP}{1\;-\;\frac{PEV}{{\text{σ}}_{\text{gi}:\text{c}}^{2}}}$$

Where PEV was the prediction error variance of the BLUP and σ_gi:c_^2^ variance of the genotypes. Deregressed BLUPs were used to perform univariate and multivariate GWAS for CBSD traits using GEMMA version 0.98.4 with default parameter settings applied (Zhou, 2012; Zhou and Stephens, 2014). The relationship matrix among clones was calculated using the *A.mat* function in the *rrBLUP* package in R (Endelman, 2012). Using the *Prcomp* function in R, principal components were determined using the relationship matrix, and these were used to account for population structure. Visualization of Manhattan, and quantile-quantile plots were implemented in the “*qqman*” R package (Turner and Turner, 2021).

### Candidate gene identification

BEDTools (Quinlan and Hall, 2010) was used to identify candidate genes in regions with GWAS hits. Identified genes were characterized for gene ontology including molecular functions, cellular components, and biological functioning using PANTHER version 17.0 (Mi et al., 2019) and *Manihot esculenta* genome version 6 gene ontology database in Phytozome (Goodstein et al., 2012). Additional gene and protein functions were also searched using Alliance of Genomes Resources (Kishore et al., 2020).

## Results

### Characterization of CBSD infections in the C2 population

CBSD mean incidence varied across years and locations, with greater incidences of 29%, 37% and 48% at 3, 6 and 12 MAP, in the 2019/2020 in Namulonge compared to 22%, 27% and 39% in Serere. The same trend was observed in the 2020/2021 growing season with even greater incidence scores. Average mean CBSD incidence in this population was 44% across the two growing seasons. CBSD mean severity for foliar symptoms also increased in both years and locations with mean severity scores of 1.4 and 1.6 at 3 and 6 MAP in the 2019/2020 in Namulonge compared to 1.3 and 1.4 in Serere respectively. Mean root severity scores were 2.1 and 1.9 in Namulonge and Serere. For the 2020/2021, mean severity scores of 1.6, 2, 2.3 were reported for Namulonge and 1.7, 1.8, 1.8 for Serere at 3, 6 and 12 MAP respectively (**Table 1**). All CBSD mean severities within and between locations across both seasons were significantly different (P ≤ 0.05). Coefficient of variation (CV) for all CBSD traits ranged from 32 - 121% with CBSD incidence scores having larger CVs.

**Table 1:** Descriptive statistics of C2 seedling and clonal evaluation trials evaluated at Namulonge and Serere in 2019/2020 and 2020/2021 seasons.

Trial	Number of plots		CBSDi3	CBSDi6	CBSDRi	CBSDs3	CBSDs6	CBSDRs
**Namulonge 2019/2020**	486	µ	28.74	36.78	47.89	1.35	1.59	2.08
		SD	34.88	8.40	30.83	0.47	0.66	0.98
		Min	0.00	0.00	0.00	1.00	1.00	1.00
		Max	100	100	100	3.00	4.00	5.00
		CV	121.35	104.42	64.38	34.97	41.20	47.02
**Serere 2019/2020**	388	µ	21.51	27.29	38.70	1.28	1.43	1.93
		SD	33.92	36.97	33.12	0.47	0.64	1.12
		Min	0.00	0.00	0.00	1.00	1.00	1.00
		Max	100	100	100	3.00	3.00	5.00
		CV	158.15	135.90	85.59	36.64	44.45	57.89
**Namulonge 2020/2021**	399	µ	50.60	62.71	–	1.59	1.99	2.28
		SD	39.95	40.23	–	0.51	0.74	1.12
		Min	0.00	0.00	–	1.00	1.00	1.00
		Max	100	100	–	3.00	3.90	5.00
		CV	79.23	64.43	–	32.23	37.11	49.17
**Serere 2020/2021**	389	µ	54.17	50.72	57.77	1.71	1.77	1.75
		SD	41.43	40.85	37.07	0.62	0.71	0.98
		Min	0.00	0.00	0.00	1.00	1.00	1.00
		Max	100	100	100	3.00	3.20	5.00
		CV	76.75	81.07	64.18	36.50	39.87	55.75
**Combined data**	1662	µ	38.40	44.12	48.46	1.48	1.69	2.00
		SD	39.97	41.26	34.52	0.55	0.71	1.06
		Min	0.00	0.00	0.00	1.00	1.00	1.00
		Max	100	100	100	3.00	4.00	5.00
		CV	104.14	93.58	71.24	37.09	42.13	52.95

µ, mean phenotype of traits; SD, standard deviation; Min, minimum value of a trait; Max, maximum value of a trait; CV, coefficient of variation(%); CBSDi3, cassava brown streak foliar incidence at 3 months after planting (MAP); CBSDi6, cassava brown streak foliar incidence at 6 MAP; CBSDs3, cassava brown streak foliar severity at 3 MAP; CBSDs6, cassava brown streak foliar severity at 3 MAP; CBSDRi, cassava brown streak root incidence; CBSDRs, cassava brown streak root severity.

### Partitioning of phenotypic variance explained by genotype, environment, and genotype-by-environment interactions

The full model had lower deviance values that were significantly different (P ≤ 0.001) from those of the reduced model for all CBSD traits (**Table 2**). There were also differences in the percentage of total phenotypic variance that was explained by genotypes. The proportion of phenotypic variance explained by genotypic variance was 66%, 64%, 23%, 62%, 66% and 55% for CBSDi3, CBSDi6, CBSDRi, CBSD3s, CBSD6s and CBSDRs respectively and this was greater than the proportion that was explained by the environment and G x E interactions which were less than 28% and 16% respectively for all CBSD traits (**Table 3**).

**Table 2:** A chi-square test comparing the deviance values for G x E model (Full Model) with a model fitted without G x E term (Reduced model).

Models					Deviance values for traits†		
	CBSDi3		CBSDi6	CBSDRi	CBSDs3	CBSDs6	CBSDRs
**Full-GxE**	15018		15989	16296	1022.4	1813.7	4386.5
**Reduced-GxE**	15192		16158	16215	1151.2	1979.9	4409.8
**Chi-sq Test**	174.18***		170.68***	83.30 ***	128.78***	166.24***	23.351***

***, significant at probability level of 0.001; and **‡**ns, non-significant; †Deviance and chi-square values for CBSDi3 = cassava brown streak foliar incidence at 3 months after planting (MAP); CBSDi6 = cassava brown streak foliar incidence at 6 MAPS; CBSDs3, cassava brown streak foliar severity at 3 MAP; CBSDs6, cassava brown streak foliar severity at 3 MAP; CBSDRi, cassava brown streak root incidence; CBSDRs, cassava brown streak root severity.

**Table 3:** Apportioning of variance components from the full model.

Traits	Proportion of variance (%) explained by Genotype, Environment and G*E		
	Genotype-by-Environment	Environment	Genotype
**CBSDi3**	14.4	9.7	65.9
**CBSDi6**	15.4	9.3	64.3
**CBSDRi**	10.2	27.8	23.4
**CBSD3s**	13.5	9.2	61.7
**CBSD6s**	14.5	7.4	66.3
**CBSDRs**	0.0	0.56	55.1

CBSDi3, cassava brown streak foliar incidence at 3 months after planting (MAP); CBSDi6, cassava brown streak foliar incidence at 6 MAP; CBSDs3, cassava brown streak foliar severity at 3 MAP; CBSDs6, cassava brown streak foliar severity at 3 MAP; CBSDRi, cassava brown streak root incidence; CBSDRs, cassava brown streak root severity.

### Broad and narrow sense heritability

Broad sense heritability estimates for CBSD traits ranged from low to high, 15% to 96% in the combined and year-location specific datasets (**Table 4**). Despite high heritability estimates, lower estimates of 0.15 and 0.40 were reported for CBSDRi and CBSDRs respectively in the Serere 2020/2021 growing season. Narrow sense heritability estimates for CBSD traits ranged from low to medium, (0.03 - 0.61) across both years and locations. The lowest estimates were reported for CBSDRi in the Serere 2019/2020 season while the highest were reported for CBSDRs in Namulonge 2019/2020 season. It was observed that both broad and narrow sense heritability estimates were higher in the 2019/2020 compared to the 2020/2021 growing season for CBSD phenotypes.

**Table 4:** Broad sense heritability of two clonal evaluation trials evaluated at Namulonge and Serere in 2019/2020 and 2020/2021 season.

Trial	CBSDi3	CBSDi6	CBSDRi	CBSDs3	CBSDs6	CBSDRs
Broad-sense heritability						
**Combined data**	0.86	0.85	0.57	0.84	0.86	0.79
**Namulonge 2019/2020**	0.92	0.95	0.89	0.89	0.96	0.86
**Namulonge 2020/2021**	0.92	0.91	–	0.86	0.91	0.63
**Serere 2019/2020**	0.95	0.94	0.66	0.90	0.95	0.63
**Serere 2020/2021**	0.96	0.94	0.15	0.96	0.90	0.40
Narrow-sense heritability						
**Combined data**	0.34	0.38	0.20	0.38	0.40	0.38
**Namulonge 2019/2020**	0.24	0.26	0.42	0.27	0.24	0.61
**Namulonge 2020/2021**	0.18	0.23	–	0.16	0.27	0.39
**Serere 2019/2020**	0.09	0.17	0.03	0.08	0.17	0.08
**Serere 2020/2021**	0.44	0.28	0.13	0.28	0.20	0.27

CBSDi3, cassava brown streak foliar incidence at 3 months after planting (MAP); CBSDi6, cassava brown streak foliar incidence at 6 MAP; CBSDs3, cassava brown streak foliar severity at 3 MAP; CBSDs6, cassava brown streak foliar severity at 3 MAP; CBSDRi, cassava brown streak root incidence; CBSDRs, cassava brown streak root severity.

### Correlation of CBSD traits

The magnitude of phenotypic and genotypic correlations varied across CBSD traits (**Table S1**). Phenotypic correlation pairs for CBSD traits in the combined dataset were high between CBSDi3, CBSDi6, CBSDs3, and CBSDs6, and all these traits had lower correlations with CBSDRi and CBSDRs. Correlations between CBSDi3, CBSDi6, CBSDs3 and CBSDs6 were significantly positive (p< 0.001) and ranged 0.70 to 0.95 while CBSDRi and CBSDRs had a significant correlation of 0.71 (p< 0.001). Both CBSDRi and CBSDRs had lower but significantly positive correlations that ranged from 0.32 to 0.46 (p< 0.001) with CBSDi3, CBSDi6, CBSDs3 and CBSDs6. Phenotypic correlations at the Namulonge and Serere experimental sites followed the same trend as previously reported in the combined datasets where CBSD foliar incidences and severities were positively significantly correlated and ranged from 0.64 to 0.96 (p< 0.001) while correlations with root incidences and severities ranged from 0.24 to 0.5 (p< 0.001) across the two seasons. Genetic correlations obtained from BLUPs in the combined dataset for CBSD foliar incidence and severity were high and ranged from 0.64 to 0.96 (p< 0.001) while correlation of these foliar traits to CBSDRi and CBSDRs were much lower and ranged from 0.28 to 0.41. This trend was also observed in location specific datasets. GEBVs obtained from SNP markers were significantly positively correlated (p< 0.001) between CBSD foliar incidence and severity traits at 3 and 6 MAP and these correlations ranged from 0.80 to 0.96 while the correlation between CBSDRi and CBSDRs was 0.83 (p< 0.001). Between CBSD foliar and root symptoms, there were positive significant correlations (p< 0.001), with values that ranged from low to moderate (0.26 to 0.55). Location- and year-specific correlations patterns did not vary from those reported in the combined dataset (**Table S2**).

### GWAS of CBSD traits in the C2 population

**Figure 1:**
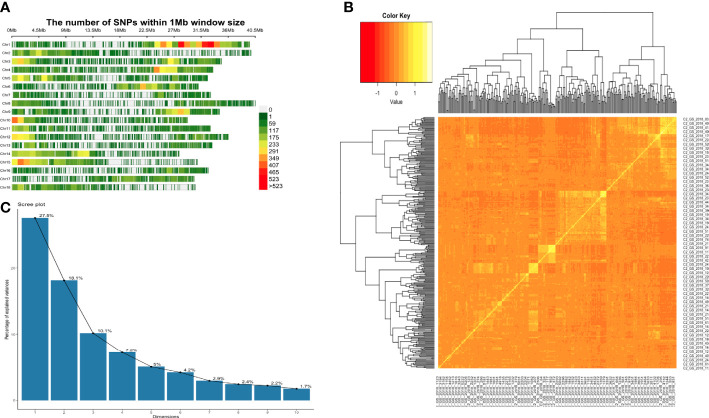
**(A)** Distribution of SNP markers across the 18 chromosomes for genotyped clones in the C2 population. The graph represents the number of SNPs within a 1 mega base window on all the 18 chromosomes in cassava: **(B)** Heatmap showing pairwise genomic relationship matrix: **(C)** The proportion of genetic variation explained by the first 10 principal components and 302 cassava clones that were in two years and two locations.

**Figure 2:**
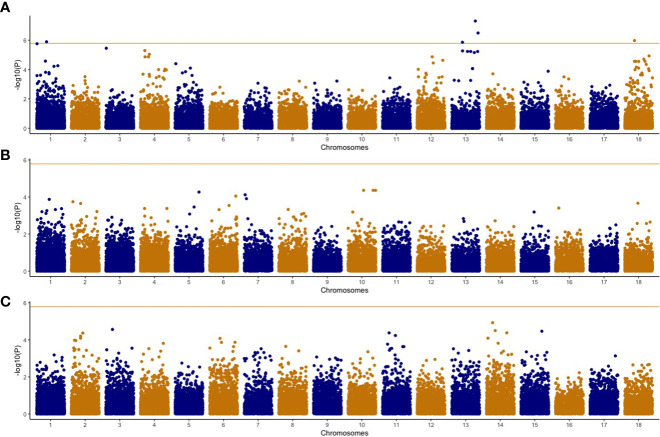
Manhattan plots of univariate genome-wide association studies for CBSD severity traits in the C2 population. **A** = cassava brown streak foliar severity at 3 MAP; **B** = cassava brown streak foliar severity at 3 MAP; **C** = cassava brown streak root severity. Orange horizontal line indicates Bonferroni genome wide significance level [-log_10_(0.05/number of markers)].

**Figure 3:**
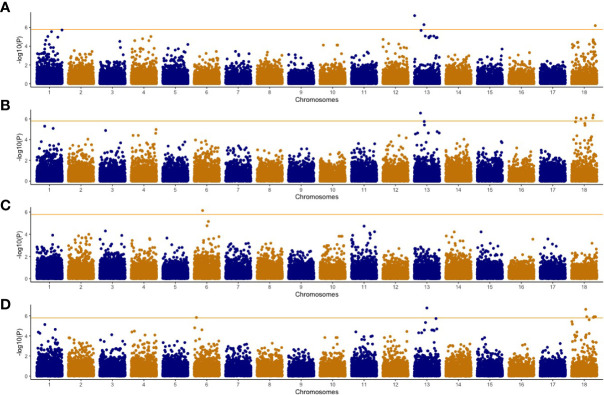
Manhattan plots of multivariate genome-wide association studies for CBSD severity trait combinations in the C2 population. **A** = cassava brown streak foliar severity at 3 and 6 MAP; **B** = cassava brown streak severity at 3 and 12 MAP; **C**= cassava brown streak severity at 6 and 12 MAP; **D** = cassava brown streak severity at 3, 6 and 12 MAP. Orange horizontal line indicates Bonferroni genome wide significance level [-log_10_(0.05/number of markers)].

We conducted a GWAS using 302 cassava genotypes and 30,846 SNP markers were used after applying filtering based on earlier described parameters. Average SNP coverage across chromosomes varied between 1,162 SNPs on chromosome 7 to 5,188 SNPs on chromosome 1 (**Figure 1A**) while average minor allele frequency was 0.24. Genomic background effects were modeled *via* a marker inferred Kinship matrix (**Figure 1B**). We also accounted for population structure using the first four principal components (PC) that explained 63% of the total phenotypic variance (**Figure 1C**). A total of 22 significant associations based on the Bonferroni threshold of 5.92 were identified across the univariate and multivariate GWAS analyses (**Table 5**). Control of population structure effects on the associations were validated using Q-Q plots where the observed -log_10_(P-value) was close to the expected -log_10_(P-value) at -log_10_(P-value)< 2.0 but at the tail of the distribution the dots deviated from the observed value thus indicating that significant associations were identified (**Supplementary Figures 2** and **3**). In the univariate analysis, 5 associations were reported only for CBSDs3 on chromosomes 1, 13 and 18 (**Figure 2**). SNPs in these three genomic regions explained 19.1% of the observed phenotypic variation compared with other SNPs in the genome that explained only 11.9%. Favorable alleles and their phenotypic effects for these SNPs are reported in **Table 6**. Furthermore, 17 associations were identified in the multivariate GWAS (**Figure 3**) for the different CBSD trait combinations and these regions were consistent with those in univariate GWAS at CBSDs3 except for the SNP on chromosome 6 (S6_3786388) that was identified between CBSDs6 and CBSDRs.

**Table 5:** Genome-wide significant markers and -log10 p-values in univariate and multivariate GWAS for CBSD severities in the C2 population.

SNP	3 MAP	3 &6 MAP	3 &12 MAP	6 &12 MAP	3, 6, &12 MAP
S1_3052885	5.89	–	–	–	–
S6_3786388	–	–	–	6.14	5.84
S13_650263	5.86	–	–	–	–
S13_1089170	6.49	6.29	–	–	–
S13_1111102	7.31	7.26	6.55	–	6.78
S18_16279925	–	–	6.09	–	5.91
S18_16447865	–	–	5.99	–	5.84
S18_18536842	–	–	6.09	–	5.91
S18_20442083	5.98	6.19	6.36	–	6.65
S18_23462935	–	–	6.09	–	5.91

**Table 6:** Proportion of variance explained (PVE), favorable SNP alleles and their phenotypic effects (PE).

Traits	SNP	PVE (%)	Position/bp	Alleles	Favorable alleles	PE	Number of plots
CBSDs3	S1_3052885	8.46	3052885	G/T	T	-1.039^‡ns^	284
	S13_650263	4.52	650263	G/T	T	-0.369^‡ns^	287
	S13_1089170	5.79	1089170	A/G	A	-16.947^***^	288
	S13_1111102	6.29	1111102	A/G	A	-2.159^‡ns^	288
	S18_20442083	8.98	20442083	G/T	G	-0.924^‡ns^	280

*** highly favorable SNP alleles that exhibit significantly different traits compared with the unfavorable alleles (P< 0.001); and **‡**ns, non-significant; CBSDs3 = cassava brown streak foliar severity at 3 MAP.

### Candidate gene identification

A total of 77 candidate genes were identified in the significant regions of 650,263 – 1,111,102 bp on chromosome 13 and 16,279,925 – 23,462,935 bp on chromosome 18 (**Table S3**). Emphasis was placed on chromosomes 13 and 18 because of their consistency between univariate and multivariate GWAS analyses. The identified genes were classified based on: (1) molecular function, (2) biological function, (3) cellular components, (4) protein groups and (5) PANTHER categories with the Manihot esculenta annotation IDs from the version 6 genome of cassava. In the molecular functions, genes were clustered into six categories namely, (1) 56.5% catalytic activity (GO.0003824), (2) 17% binding (GO:0005488), (3) 13% transporter activity (GO:0005215), (4) 4.3% ATP-dependent activity (GO:0140657), (5) 4.3% translation regulator activity (GO:0045182), and (6) 4.3% molecular function regulator (GO:0098772). In biological classification, eight groups were identified that included (1) 37% cellular process (GO:0009987), (2) 23.9% metabolic process (GO:0008152), (3) 15.2% response to stimulus (GO:0050896), (4) 8.7% biological regulation (GO:0065007), (5) 6.5% localization (GO:0051179), (6) 4.3% signaling (GO:0023052), (7) 2.2% development process (GO:0032502), and (8) 2.2% multicellular organismal process (GO:0032501). Classification of the cellular components identified two entities, 90% cellular anatomical entity (GO:0110165) and 9.1% protein-containing complex (GO:0032991), while twelve protein classes were identified based on the protein classification system. These included (1) 25.8% metabolite interconversion enzyme (PC00262), (2) 19.4% protein modifying enzyme (PC00260), (3) 12.9% DNA metabolism protein (PC00009), (4) 9.7% gene-specific transcriptional regulator (PC00264), (5) 6.5% transporter (PC00227), (6) 6.5% translational protein (PC00263), (7) 3.2% RNA metabolism protein (PC00031), (8) 3.2% chaperone (PC00072), (9) 3.2% chromatin/chromatin-binding, or regulatory protein (PC00077), (10) 3.2% cytoskeletal protein (PC00085), (11) 3.2% membrane traffic protein (PC00150), and (12) 3.2% scaffold/adaptor protein (PC00226). Finally, the PANTHER pathway identified three categories, (1) 33.3% apoptosis signaling pathway (P00006), (2) 33.3% transcription regulation by bZIP transcription factor (P00055), and (3) 33.3% ubiquitin proteasome pathway (P00060).

## Discussion

We characterized the C2 population for cassava brown streak disease using the 1-5 visual scoring method. Both univariate and multivariate GWAS were conducted to find genomic regions associated with CBSD traits, followed by the identification of annotated genes in these genomic regions and their functions. Results revealed that CBSD incidence and severity increased between foliar severities at 3 and 6 MAP across the two growing seasons and locations. CBSD root incidence and severity also increased across all environments. It was observed that Namulonge had higher incidence and severity scores compared to Serere as previously reported (Kaweesi et al., 2014; Ogwok et al., 2015; Anthony et al., 2015), substantiating that Namulonge remains a CBSD hotspot. Observed variation in CBSD phenotypes in the C2 population was mainly due to genetic effects, which differed from previous studies where variation was largely due to environmental effects (Nduwumuremyi et al., 2017; Shirima et al., 2020). Variation in CBSD phenotypes in the C2 population can be attributed to directional cyclic selection of clones using genomic selection that relies on estimated breeding values that reflect the actual performance of progeny (Meuwissen et al., 2001).

Limited influence of environment and genotype by environment interactions can explain the moderate to high broad-sense heritability observed for CBSD incidence and severity across environments as earlier reported (Okul Valentor et al., 2018). Progressive increase in heritability estimates between cycles of genomic selection was previously reported with estimates increasing from C0 to C1 population (Ozimati et al., 2019). The nature of the observed heritability further reinforces that the variation in CBSD traits was largely due to genetics rather than environmental effects. There were also differences in CBSD estimates across the two evaluation years that were reported in our study and this could typically be explained by contrasting weather conditions and whitefly population densities (Okul Valentor et al., 2018). We also hypothesize that systemic infections that arise from accumulation of viral load/titer due to continual recycling of stem cutting across years (Kaweesi et al., 2014) could be responsible for the difference in estimates across years. This hypothesis requires further investigation. The observation on low to moderate narrow sense heritability was similar to a previous study (Ozimati et al., 2019) with lower estimates compared to the broad sense estimates, and this was attributed to varying levels of linkage disequilibrium between markers and the causal loci. In our marker dataset, the density of SNP markers varied across different chromosomes (**Figure 1A**) which could have caused uneven LD between SNPs and causal loci leading to under representation of narrow sense heritability estimates. The difference can also be attributed to the amount of non-additive variation that is captured in broad sense heritability estimates but not in narrow sense heritability estimates.

We also calculated correlations between phenotypic estimates, genetic estimates, and genomic estimated breeding values. It was observed that there were positive phenotypic and genetic correlations between CBSD traits. Comparable observations for high phenotypic (Rwegasira and Rey, 2012; Ozimati et al., 2019) and genetic correlations (Ozimati et al., 2019) were previously reported for CBSD in Uganda and Tanzania. These moderate to high correlations can be leveraged to reduce costs in the breeding process *via* indirect selection. The practical implication would be that CBSD foliar symptoms scoring at 6 MAP would reflect the clone’s performance at 3MAP. So, only phenotyping at 6 MAP would reduce phenotyping costs. Genetic and GEBV correlations in our study were also high and can be attributed to gene actions like linkage disequilibrium and pleiotropy that create genetic correlations thus creating a dependence between traits (Walsh and Blows, 2009) which can still be leveraged in breeding as earlier mentioned.

Genome wide association mapping is a powerful tool that has been used in numerous crops to investigate the genetic architecture of complex traits, including plant diseases. Genomic regions/causative loci that confer either resistance/susceptibility to various pathogens (Bartoli and Roux, 2017) have been identified, and these have played a role in developing markers for marker assisted selection. Our GWAS study was made up of clones that were genetically diverse with low stratification in the principal components (**Supplementary Figure 1**), leading to the detection of 22 significant associations that were distributed on chromosome 1, 13, and 18 from both univariate and multivariate analyses (**Figures 2**, **3**). The proportion of phenotypic variance explained by these significant SNPs was 19%, an indicator that the effects were not from a major gene. A comparable observation was reported earlier with the highest SNP effects identified on chromosome 11 from GWAS analyses conducted on two cassava panels that consisted of 429 and 872 clones, explaining only 6% of the phenotypic variance (Kayondo et al., 2018). Previous GWAS and QTL studies on CBSD using the 1-5 scoring method have identified multiple regions associated with CBSD traits. A recent study identified two genomic regions on chromosome 4 and 11 that were associated with CBSD foliar severities at 3 and 6 MAP while no associations were identified for CBSD root severity (Kayondo et al., 2018). Furthermore, 9 QTLs from a biparental population that were located on chromosomes 4, 5, 6, 11, 12, 15, 17, and 18 were reported for cassava in Tanzania (Nzuki et al., 2017). QTLs on chromosomes 4, 6, 17 and 18 were associated with CBSD foliar symptoms while those on chromosomes 5 and 12 were associated with CBSD root necrosis. Only two QTLs on chromosomes 11 and 15 were associated with both CBSD foliar and root symptoms. Also, three QTLs on chromosomes 2, 11 and 18, associated to CBSD foliar and root symptoms were reported, including 27 genes identified on chromosome 18 (Masumba et al., 2017). These genes included LRR proteins and signal recognition particles. Finally, seven significant SNP markers on chromosome 11, associated with mean root severity and disease index were reported for CBSD in Uganda (Kawuki et al., 2016). Our study identified marker SNPs on chromosomes 6 and 18 that were comparable to previous observations (Nzuki et al., 2017; Masumba et al., 2017). It was reported that chromosome 18 had an introgression region from *Manihot glaziovii* in the Kiroba clone that was a progenitor in the biparental population that identified region associated with CBSD on chromosome 18 (Nzuki et al., 2017). It was also reported that the region on chromosome 18 contained the F-box domain with LRR domains and the pentatricopeptide repeat (PPR) superfamily proteins that are associated with pathogen response.

Despite the similarity between our genomic regions and those from earlier studies (Nzuki et al., 2017; Masumba et al., 2017), the differences with other studies for CBSD in Uganda (Kawuki et al., 2016; Kayondo et al., 2018) cannot be overlooked. This is because many genotypes evaluated in these studies (Kawuki et al., 2016; Kayondo et al., 2018) are progenitors of the C2 population. This observation can be attributed to differences in allelic architecture (number of distinct alleles that affect disease susceptibility at a given locus) or linkage disequilibrium across the different populations that could have been exacerbated by recurrent selection leading to genetic drift. This could have caused a shift in allele frequency between the C2 population and other populations from Uganda. The same difference can also be attributed to the Bulmer effect that shrinks the proportion of genetic variance which arises from selection (Tallis, 1987). Furthermore, we postulate that alleles that control CBSD resistance or susceptibility could be rare, making it difficult to identify them through GWAS or even in biparental populations despite using large sample sizes and correcting for population structure (Wang et al., 2016; Wen et al., 2019). Genetic diversity of a population also influences the probability of mining rare alleles (Fu, 2015). A look into the pedigrees of the C2 population (**Table S4**) showed that there was limited diversity in the parents (117), grandparents (65), and great grandparents (13) of this population. To put this concept of limited diversity into perspective, 41% of the grandparents and 69% of the great grandparents of the C2 population were used two or more times as progenitors. This means that if rare alleles are responsible for CBSD, there is a possibility that they are not captured during hybridization and selection. And this could be responsible for weak associations (Gudbjartsson et al., 2007; Helgason et al., 2007; Yamada et al., 2009), leading to detection of different regions associated to CBSD in different populations (Lewis et al., 2008). Therefore, we propose conducting a meta-analysis for all CBSD trials conducted in East and Central Africa. Such a study would improve the chances of identifying genes including rare alleles with larger effects, while leveraging populations that are phenotyped in multiple environments with varying CBSD pressure. In addition, we propose expanding CBSD phenotyping methods to include virus titer quantification (Kaweesi et al., 2014) and root necrosis image analysis (Tusubira et al., 2020). These proposed studies are currently on-going and will expand our understanding of the genetic architecture of CBSD foliar and root traits in cassava.

Functions of annotated genes characterized in our GWAS regions of interest include (1) hydrolyzing ATP, (2) interacting with molecules, (3) catalyzing reactions, (4) initiating, activating, perpetuating, or terminating polypeptides synthesis in ribosomes and (5) directed movement of molecules between and within cells. Protein functions identified include (1) modifying DNA, (2) processing and metabolizing RNA, (3) unfolding polypeptides, (4) binding chromatin, (5) forming flexible frameworks for cells to provide attachment points and (6) communication between cells. In addition to regulating transcription of specific sets of genes, docking or fusion of vesicle to cytoplasmic membrane, conversion of small molecules to other forms, covalent modification of proteins, and translation of mRNA to proteins. Three pathway groups were also identified based on the PANTHER classification system that were shown to induce targeted degradation by proteasome machinery thus regulating various protein functions for virus replication and pathogenesis (Zhou and Zeng, 2017; Dubiella and Serrano, 2021). The mechanism of targeted degradation by proteasome machinery has been extensively studied in potato virus Y (PVY) (Jin et al., 2007). Thus, the identified genes, proteins and pathways may play critical roles in biological processes that enhance disease responses in plants. It is not a surprise that these proteins have been associated with CBSD severity scores at 3MAP because this stage is critical in disease establishment and advancement. Just like other viruses, CBSVs have small genomes which increases their dependence on host genes and pathways to complete the infection lifecycle (Garciá and Pallás, 2015; Wan et al., 2015; Nagy, 2016; Hyodo and Okuno, 2016; Leisner and Schoelz, 2018; Garcia-Ruiz, 2018). This can explain why numerous genes, proteins and pathways identified in our study are associated with virus establishment, replication, cell to cell movement and transmission. We hypothesize that these genes, proteins, and pathways may play roles in enhancing susceptibility of clones to CBSD, but further studies are needed to test this hypothesis.

## Conclusions

This study characterized CBSD in the C2 population. Genotypes explained a large proportion of phenotypic variance with little influence from the environment and genotype by environment interactions. This makes this population a great resource for association mapping. Heritability and correlation estimates were positive and ranged from moderate to high. Observed heritability could be leveraged to reduce costs in the breeding process through indirect selection mainly for CBSD foliar symptoms. This study also identified three genomic regions in univariate analysis on chromosomes 1, 13, and 18 and these were linked to CBSD foliar severity at 3MAP and annotated genes in these regions have been shown to enhance susceptibility to disease. These regions were consistent both in univariate and multivariate GWAS. Identification of these associations is a first step towards pinpointing SNP markers/genomic regions that could be leveraged in developing markers that will be used in marker assisted selection or genomic selection to improve selection efficiency for cassava brown streak disease breeding, thus enhancing food and economic security in Sub Saharan Africa.

## Data availability statement

The datasets presented in this study can be found in online repositories. The names of the repository/repositories and accession number(s) can be found below: https://cassavabase.org/, https://cassavabase.org/breeders/trial/6707, https://cassavabase.org/breeders/trial/7071, https://cassavabase.org/breeders/trial/7795, https://cassavabase.org/breeders/trial/7746.

## Author contributions

LN and KR conceived and designed the study. MK and KR collected data. LN and AO performed data analysis. LN wrote the manuscript. LN, AO, KR, MK, and J-LJ reviewed and revised the manuscript. All authors contributed to the article and approved the submitted version.

## Supplementary material

Supplementary Figure 1 Plot of the first four principal components (PCs) of the cycle two population.

Supplementary Figure 2 QQ plots of univariate genome-wide association studies for CBSD severity traits in the C2 population. **A** = cassava brown streak foliar severity at 3 MAP; **B** = cassava brown streak foliar severity at 3 MAP; **C** = cassava brown streak root severity.

Supplementary Figure 3 QQ plots of multivariate genome-wide association studies for CBSD severity trait combinations in the C2 population. **A** = cassava brown streak foliar severity at 3 and 6 MAP; **B** = cassava brown streak severity at 3 and 12 MAP; **C**= cassava brown streak severity at 6 and 12 MAP; **D** = cassava brown streak severity at 3, 6 and 12 MAP.
