# Supplementary figures and images for: Genetic dissection of cassava brown streak disease in a genomic selection population
Source: Front Plant Sci. 2023 Jan 13;13:1099409. doi: 10.3389/fpls.2022.1099409 (PMC9880483; doi:10.3389/fpls.2022.1099409)

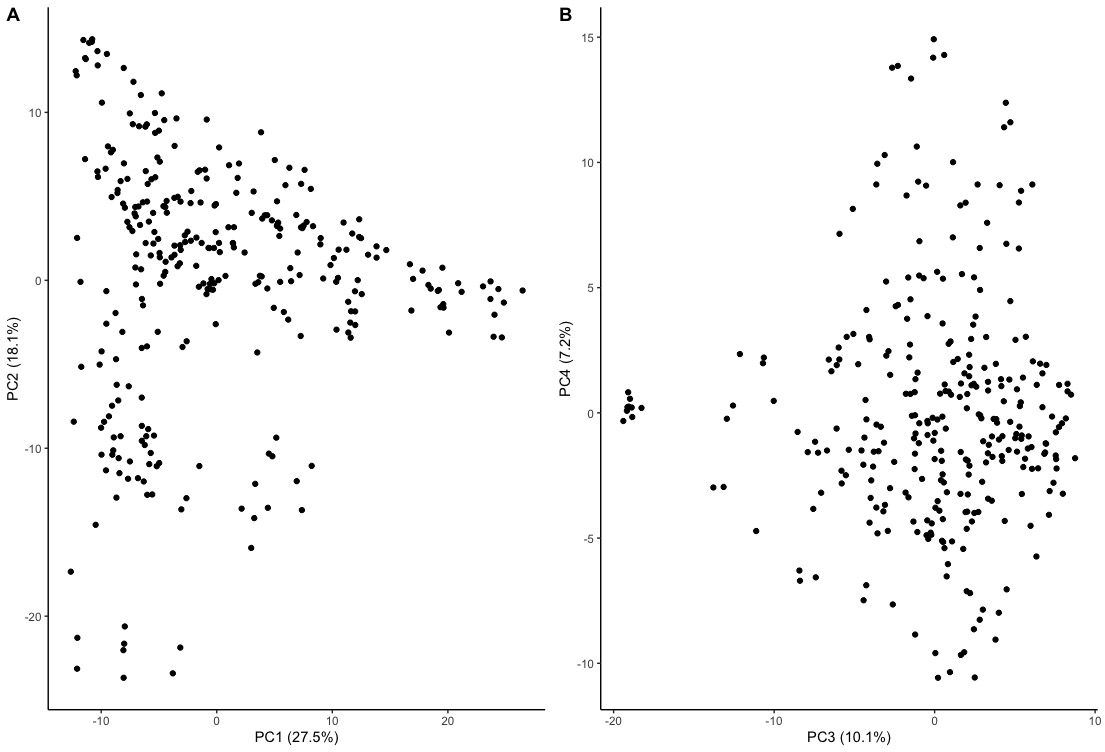

Supplement: Supplementary Figure 1 — Plot of the first four principal components (PCs) of the cycle two population. [file Image_1.jpeg]

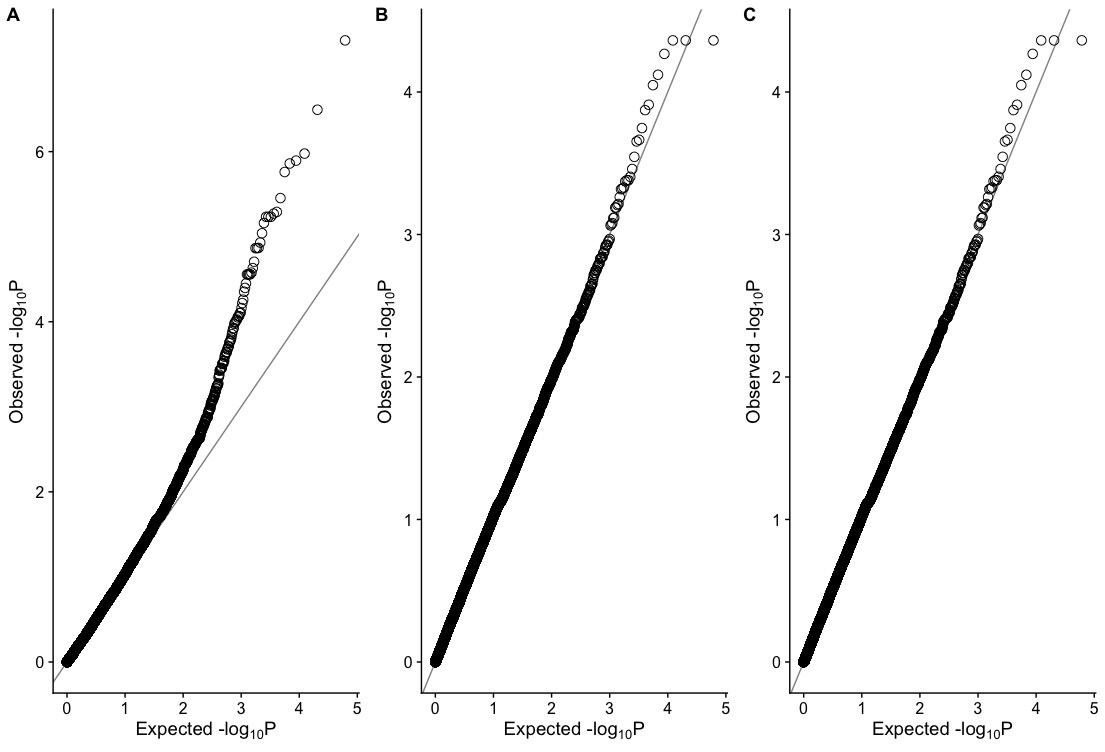

Supplement: Supplementary Figure 2 — QQ plots of univariate genome-wide association studies for CBSD severity traits in the C2 population. A = cassava brown streak foliar severity at 3 MAP; B = cassava brown streak foliar severity at 3 MAP; C = cassava brown streak root severity. [file Image_2.jpeg]

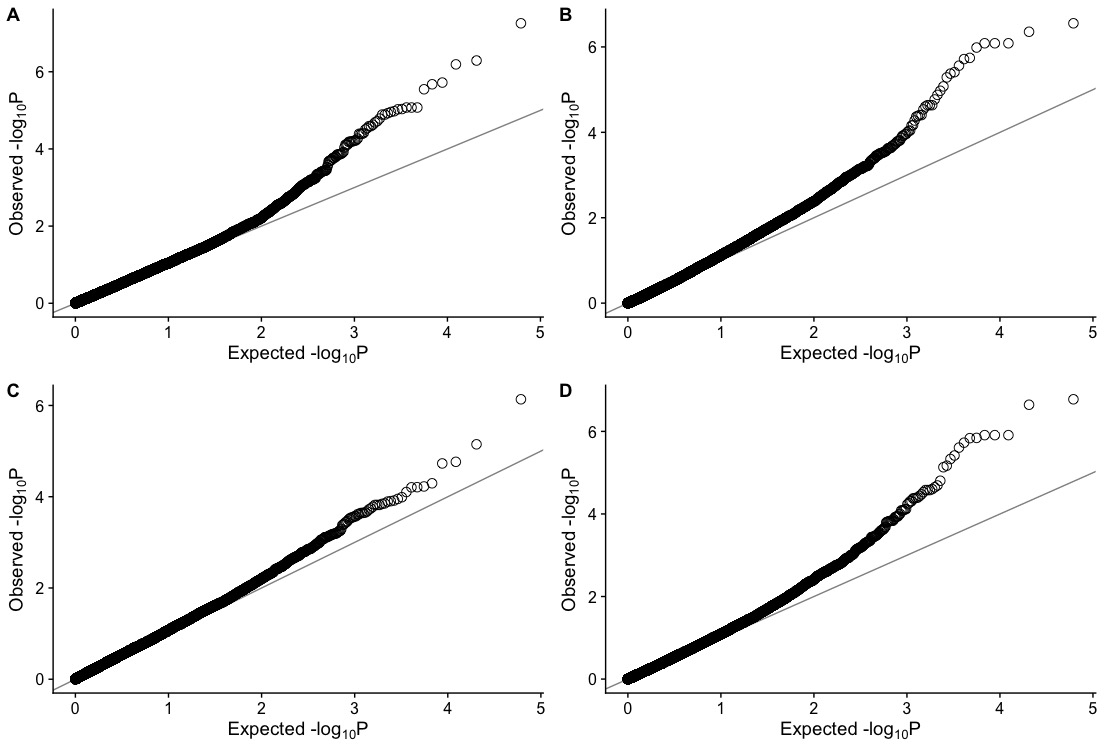

Supplement: Supplementary Figure 3 — QQ plots of multivariate genome-wide association studies for CBSD severity trait combinations in the C2 population. A = cassava brown streak foliar severity at 3 and 6 MAP; B = cassava brown streak severity at 3 and 12 MAP; C= cassava brown streak severity at 6 and 12 MAP; D = cassava brown streak severity at 3, 6 and 12 MAP. [file Image_3.jpeg]
